# Active autophagy but not lipophagy in macrophages with defective lipolysis

**DOI:** 10.1016/j.bbalip.2015.06.005

**Published:** 2015-07-02

**Authors:** Madeleine Goeritzer, Nemanja Vujic, Stefanie Schlager, Prakash G. Chandak, Melanie Korbelius, Benjamin Gottschalk, Christina Leopold, Sascha Obrowsky, Silvia Rainer, Prakash Doddapattar, Elma Aflaki, Martin Wegscheider, Vinay Sachdev, Wolfgang F. Graier, Dagmar Kolb, Branislav Radovic, Dagmar Kratky

**Affiliations:** a Institute of Molecular Biology & Biochemistry, Center of Molecular Medicine, Medical University of Graz, Harrachgasse 21, 8010 Graz, Austria; b Center for Medical Research/Institute of Cell Biology, Histology and Embryology, Medical University of Graz, Harrachgasse 21, 8010 Graz, Austria

**Keywords:** Adipose triglyceride lipase, Hormone-sensitive lipase, Lipid droplets, Triglyceride mobilization

## Abstract

During autophagy, autophagosomes fuse with lysosomes to degrade damaged organelles and misfolded proteins. Breakdown products are released into the cytosol and contribute to energy and metabolic building block supply, especially during starvation. Lipophagy has been defined as the autophagy-mediated degradation of lipid droplets (LDs) by lysosomal acid lipase. Adipose triglyceride lipase (ATGL) is the major enzyme catalyzing the initial step of lipolysis by hydrolyzing triglycerides (TGs) in cytosolic LDs. Consequently, most organs and cells, including macrophages, lacking ATGL accumulate TGs, resulting in reduced intracellular free fatty acid concentrations. Macrophages deficient in hormone-sensitive lipase (*H0*) lack TG accumulation albeit reduced in vitro TG hydrolase activity. We hypothesized that autophagy is activated in lipase-deficient macrophages to counteract their energy deficit. We therefore generated mice lacking both ATGL and HSL (*A0H0*). Macrophages from *A0H0* mice showed 73% reduced neutral TG hydrolase activity, resulting in TG-rich LD accumulation. Increased expression of cathepsin B, accumulation of LC3-II, reduced expression of p62 and increased DQ-BSA dequenching suggest intact autophagy and functional lysosomes in *A0H0* macrophages. Markedly decreased acid TG hydrolase activity and lipid flux independent of bafilomycin A1 treatment, however, argue against effective lysosomal degradation of LDs in *A0H0* macrophages. We conclude that autophagy of proteins and cell organelles but not of LDs is active as a compensatory mechanism to circumvent and balance the reduced availability of energy substrates in *A0H0* macrophages.

## 1. Introduction

Macroautophagy (hereafter autophagy) is a lysosomal pathway that degrades superfluous or damaged organelles as well as cytoplasmic inclusions such as misfolded protein aggregates [1]. These cytoplasmic cargos are trapped inside double-membrane vesicles (autophagosomes) that ultimately fuse with lysosomes for degradation of their contents. Subsequently, the breakdown products are released into the cytosol and contribute to energy and metabolic building block supply, particularly in times of starvation. FAs as energy substrates can be released through the autophagic degradation of cytosolic LDs in the lysosome by lysosomal acid lipase (LAL) (a process termed lipophagy) [2] or through intracellular lipolysis by breakdown of TGs. Thus, along with lipolysis, autophagy is one of two conserved responses to fasting. The role of autophagy in lipid metabolism is considered to be highly tissue- and condition-dependent. In the liver, autophagy may contribute to lipolysis under a high-fat diet or prolonged fasting. Autophagy might also promote lipid accumulation under normal fasting conditions [2]. Interestingly, ablation of autophagy-related 7 (ATG7) in adipose tissue resulted in lean mice with reduced white but increased brown adipose tissue mass, enhanced insulin sensitivity, and elevated β-oxidation [3]. The authors argued that inhibition of white adipocyte differentiation may lead to a defect in lipogenesis or that blocked autophagy promotes transdifferentiation from white to brown fat [3]. Zechner and colleagues hypothesized that in adipose tissue autophagy might be involved in adipocyte differentiation and lipogenesis but not in lipolysis [4]. In mouse embryonic fibroblasts, acute nutrient starvation was ineffective to induce lipophagy, whereas serum depletion in the presence of amino acids and glucose triggered lipophagy [5].

The role of autophagy in the breakdown of lipids in lipolytically active macrophages has not been investigated so far. Macrophages have an enormous capacity to store lipids within LDs. Uncontrolled accumulation of cholesteryl esters (CE), however, results in macrophage-derived foam cell formation, which is the initial stage of atherosclerosis[6,7]. Both FAs and FC are mobilized from LDs by intracellular lipases. Neutral TG hydrolysis is mainly catalyzed by adipose triglyceride lipase (ATGL), which specifically cleaves TGs [8–10]. Hormone-sensitive lipase (HSL) exhibits broad substrate specificity with highest activities toward diacylglycerol (DG) and CE followed by TG and monoacylglycerol [11–13]. Accumulation of DG but not TG in tissues of *Hsl-deficient* (*H0*) mice argued against an important role of HSL as TG hydrolase in mice [12]. Comparable with other cells and tissues, *H0* macrophages lack TG accumulation albeit reduced in vitro TG hydrolase activity [14]. Massive accumulation of TGs in essentially all cells and organs of *Atgl-deficient* (*A0*) mice demonstrated that ATGL plays a crucial role in lipolysis [15]. *A0* mice have a short life span and die at the age of 12–14 weeks due to severe cardiomyopathy [15]. Knockdown of HSL in *A0* white adipose tissue resulted in more than 95% reduction of TG hydrolase activity [16], indicating that ATGL and HSL are the major enzymes in TG catabolism of adipose tissue. We have recently shown that ATGL plays an important role in TG hydrolysis in macrophages and its absence markedly impairs phagocytic capacity [17] and macrophage migration [18], induces the mitochondrial apoptosis pathway [19], and ER stress [20]. Transfer of *A0* bone marrow into LDL receptor-deficient mice attenuated atherosclerotic lesion development compared to wild-type (Wt) bone marrow-transplanted animals [21], suggesting that ATGL deficiency in myeloid cells (including macrophages) has anti-atherosclerotic properties.

To investigate the contribution of ATGL and HSL to TG hydrolysis in murine macrophages, we generated *A0H0* mice. We hypothesized that macrophages lacking ATGL and HSL have defective lipolysis but active autophagy to circumvent the lack of FAs for energy production. Our data demonstrate markedly reduced neutral TG hydrolase activity, resulting in TG-rich LD accumulation in *A0H0* macrophages. In addition, we provide evidence that autophagy is a consistently active process in macrophages, including lipase-deficient cells. Notably, our results indicate that degradation of LDs by lysosomal acid lipase (LAL) is likely not an adaptive mechanism for generating FAs as energy substrate in *A0H0* macrophages.

## 2. Materials and methods

### 2.1. Animals

*A0* (due to their short life span) [15] and male *H0* (infertile) mice [12] cannot be used for breeding. Therefore, *Atgl+/−* mice were bred with female *H0* mice to create *Atgl+/−Hsl+/−* mice, which were then crossed to generate *A0H0* mice. Mice were kept on chow diet (containing 4% fat and 19% protein; Altromin Spezialfutter GmbH, Lage, Germany) and water ad libitum on a regular light–dark cycle (12 h/12 h). The following primers were used for genotyping: HSL-forward 5′-CATGCACCTAGTGCCATCCTTC-3′; HSL-reverse 5′-CTCACTGAGGCC TGTCTCGTTG-3; ATGL-forward 5′-AGAGAGAGAAGCTGAAGCCTG-3′, ATGL-reverse 5′-GCCAGCGAATGAGATGTTCC-3′. Animal experiments were performed according to the standards set by the Austrian Federal Ministry of Science and Research, Division of Genetic Engineering and Animal Experiments, Vienna, Austria.

### 2.2. Macrophage lipid parameters

Mouse peritoneal macrophages were collected after an i.p. injection of 2 ml 3% thioglycolate broth. After 3 days, peritoneal cells were collected by peritoneal lavage using 10 ml PBS/EDTA. Macrophages were cultured in serum-free DMEM (Gibco®, Life Technologies, Carlsbad, CA) for 2 h. Thereafter, cells were washed 3 times with pre-warmed PBS and lipids were extracted by the addition of 2 ml hexane:isopropanol (3:2, v:v) for 2 h at 4 °C. The lipid extract was dried under a stream of nitrogen. One hundred microliters 1% TritonX-100 in chloroform were added, samples were vortexed and dried again under nitrogen. Thereafter, the samples were resuspended in 100 μl ddH_2_O, and TG and TC concentrations were measured enzymatically by commercially available kits (DiaSys, Holzheim, Germany). Results were normalized to protein concentrations after complete cell lysis with 2 ml 0.3 M NaOH/well for 2 h at RT.

### 2.3. TG and CE hydrolase activity assays

Macrophages were cultured in DMEM containing 10% lipoprotein-deficient serum (LPDS) and 1% penicillin/streptomycin (P/S) for 2 h. Thereafter, cells were washed twice with pre-warmed PBS and adherent cells (macrophages) were cultured in DMEM/10% LPDS and 1% P/S for 24 h. For measuring acid TG and CE hydrolase activities, macrophages were lysed with 200 μl citrate lysis buffer containing 250 mM sucrose, 1 mM EDTA, 0.1 mM DTT (pH 4.5), and sonicated on ice four times for 30 s with 30 s interval, Protein concentrations were measured using a Lowry assay. Preparation of TG and CE substrates as well as determination of neutral TG and CE hydrolase activities have been recently published [22].

### 2.4. Western blotting

Protein samples (40 μg protein/lane) of lysed macrophages from the different genotypes were separated by 15% SDS-polyacrylamide gel electrophoresis and transferred to polyvinylidene difluoride or nitrocellulose membranes. Blots were incubated with anti-rabbit polyclonal antibodies against ATGL (1:200), HSL (1:800), microtubule-associated protein light chain 3 (LC3B) (1:1000), p62 (1:1000), cathepsin B (1:1000) (all purchased from Cell Signaling Technology, Danvers, MA), LAL (1:500; Seven Hills Bioreagents, Cincinnati, OH), and a monoclonal anti-mouse β-actin antibody (1:7000) (purchased from Santa Cruz, Heidelberg, Germany). The horseradish peroxidase-conjugated goat anti-rabbit (1:2000) and rabbit anti-mouse antibodies (1:1000) (Dako, Glostrup, Denmark) were visualized by enhanced chemiluminescence detection (ECL Plus; Thermo Scientific, Rockford, IL or Clarity^™^ Western ECL Substrate; Bio-Rad, Austria) using the BioRad ChemiDoc^™^ MP Imaging System (BioRad Laboratories Inc., Hercules, CA) or AGFA Curix Ultra X-Ray films (Siemens, Graz, Austria).

### 2.5. Nile red staining

Neutral lipids were visualized by Nile red staining as described [17]. Images were captured by confocal laser scanning microscopy using an LSM 510 META microscope system (Carl Zeiss GmbH, Vienna, Austria). Quantification of LDs was performed in at least 300 cells per group using Zeiss LSM Image Browser (Carl Zeiss Microimaging GmbH, Vienna, Austria).

### 2.6. Autophagic flux and lysosomal function analyses

Macrophages were incubated with bafilomycin A1 (10 nM; Sigma-Aldrich, Vienna, Austria) for 0 and 14 h for protein isolation and Western blotting experiments using anti-p62 and anti-LC3 antibodies.

In addition, DQ^™^ Red BSA (DQ-BSA; Molecular Probes, Eugene, OR), a self-quenched red BODIPY dye conjugated to BSA, was used for measuring lysosomal activity. DQ-BSA requires enzymatic cleavage in acidic intracellular lysosomal compartments to generate a fluorescent product that can be monitored by flow cytometry and fluorescence microscopy. Macrophages were cultured in DMEM/10% LPDS/1% penicillin/streptomycin for 24 h. To induce autophagy, cells were fasted for 1 h in Hank’s Balanced Salt Solution (HBSS; PAA Laboratories, Linz, Austria). Cells were then incubated with DQ-BSA (final concentration: 10 μg/ml) for 15 min at 37 °C. After washing the cells twice with PBS, macrophages were cultured in DMEM/10% LPDS, and samples were taken after 0, 2, and 6 h, respectively. Red-fluorescent DQ-BSA was analyzed by flow cytometry using a FACSCalibur flow cytometer (BD Biosciences, Mountain View, CA).

### 2.7. Oleate uptake

Macrophages were plated in 12-well plates and incubated with 300 μM OA-BSA and 1 μCi of [^3^H]OA-BSA in DMEM/1% P/S per well for 24 h. Cells were washed twice with PBS and then equilibrated in DMEM/1% P/S for 1 h. Thereafter, macrophages were cultivated in DMEM/1% P/S containing 2% FA-free BSA in the absence and presence of Atglistatin (Ai; 40 μM), HSL inhibitor (Hi; 25 μM) [23], and bafilomycin A1 (10 nM). After 2, 6 or 14 h, an aliquot of medium was taken for determination of FA release. After 14 h, lipids were isolated from cells by hexane:isopropanol (3:2) extraction for 1 h. The extract was dried under liquid nitrogen and dissolved in 50 μl of human serum. Each sample was loaded on a TLC plate and separated using hexane:diethyl ether:acetic acid (70:30:1, v:v:v) as solvent. The incorporated radioactivity was determined by liquid scintillation counting. Cells were lysed in 1 ml of 0.3 M NaOH and protein content was quantitated by Lowry assay.

### 2.8. Immunofluorescence

Macrophages were plated on coverslips in 12-well plates in DMEM/10% LPDS/1%P/S containing Bodipy ® 500/510 C1, C12 (C12-BODIPY; 0.4 μg/ml, Life Technologies) for 24 h. After incubation, cells were washed twice with PBS and then equilibrated in DMEM/1% P/S for 1 h. Thereafter, cells were washed and incubated in HBSS and bafilomycin A1 (10 ng/ml) for 1 h to chase incorporated C12-BODIPY. After rinsing, cells were fixed with 4% paraformaldehyde for 10 min at RT. Cells were washed three times for 5 min with PBS. Permeabilization was then performed with 0.25% Triton X-100 in PBS for 10 min at RT followed by washing three times for 5 min with PBS. Subsequently, cells were blocked with 1% BSA in glycine/PBS for 30 min at RT and incubated with anti-cathepsin D primary antibody (1:1200; Abcam, Cambridge, UK) in 1% BSA/PBS at 4 °C overnight. Macrophages were washed three times with PBS for 5 min and incubated with secondary antibody (1:250, goat anti-mouse IgG (H + L) Alexa Fluor® 594 conjugated, Life Technologies) for 1 h at RT. Afterwards, cells were washed three times for 5 min with PBS and nuclear staining was performed with Hoechst. After washing (three times for 5 min with PBS), slides were mounted in Dako fluorescent mounting medium (Dako Denmark A/S, Glostrup, Denmark). Confocal images were acquired with a Zeiss Observer Z.1 inverted microscope (Carl Zeiss Microimaging GmbH, Goettingen, Germany) equipped with a Yokogawa CSU-X1 Nipkow spinning disk system (via Visitron Systems GmbH, Puchheim, Germany), a piezoelectric z-axis motorized stage (CRWG3-200, Nippon Thompson Co., Ltd, Tokyo, Japan), and a CoolSNAP HQ2 CCD Camera (Photometrics, Tucson, AZ). Fixed cells were excited with 405 nm, 488 nm and 568 nm laser lines (Visitron Systems GmbH) with exposure times of 2000, 500 and 600 ms using an alpha Plan-Fluor 100×/1.45 Oil M27 objective (Carl Zeiss Microimaging). Images were automatically background corrected and blind-deconvolved using Huygens 2.4.1 (Scientific Volume Imaging, SVI, VB Hilversum, The Netherlands). Colocalization analysis was performed with the ImageJ plugin JACOP [24].

### 2.9. Electron microscopy

Transmission electron microscopy of macrophages was performed as recently described [22]. Briefly, macrophages were cultured on an Aclar film, fixed in 0.1 M phosphate buffer (pH 7.4) containing 2.5% glutaraldehyde and 2% formaldehyde (2 h), post-fixed in 2% OsO_4_ (2 h), dehydrated in graded series of ethanol, and embedded in a TAAB epoxy resin.

For high-pressure freezing (HPF), macrophages were grown on carbon-coated sapphire discs and frozen under liquid nitrogen conditions using 2000 bar within ms. Freezing was followed by freeze substitution in acetone by adding 2% OsO_4_ and 0.2% uranyl acetate at temperatures below −70 °C. After water in form of ice within the cells was replaced by substitution media, samples were embedded in epoxy resin.

Images of 75 nm sections (stained with lead citrate and uranyl acetate) were taken on a FEI Tecnai G2 20 transmission electron microscope (FEI, Eindhoven, Netherlands) with a Gatan ultrascan 1000 CCD camera (acceleration voltage 120 kV).

### 2.10. Statistics

Statistical analyses were performed using GraphPad Prism 5.0 software. The significance was determined by Student’s unpaired t-test relative to controls, including Welch’s corrections in cases of unequal variances. Data are presented as mean + SD or SEM. Significance levels were set at p < 0.05 (*), p ≤ 0.01 (**) and p ≤ 0.001 (***).

## 3. Theory

Macrophages from *A0* and *H0* mice show markedly reduced neutral TG hydrolase activity. ATGL and HSL were reported to be the major enzymes in adipose tissue TG metabolism. To investigate the contribution of ATGL and HSL to TG hydrolysis in murine macrophages, we generated *A0H0* mice and determined whether additional lipase(s) might be involved in the catalytic breakdown of TG in macrophages. To circumvent the lack of FAs for energy production, we expected that macrophages lacking ATGL and HSL induce autophagy.

## 4. Results

### 4.1. Neutral TG hydrolase activity is abolished in A0H0 macrophages

**Fig. 1:**
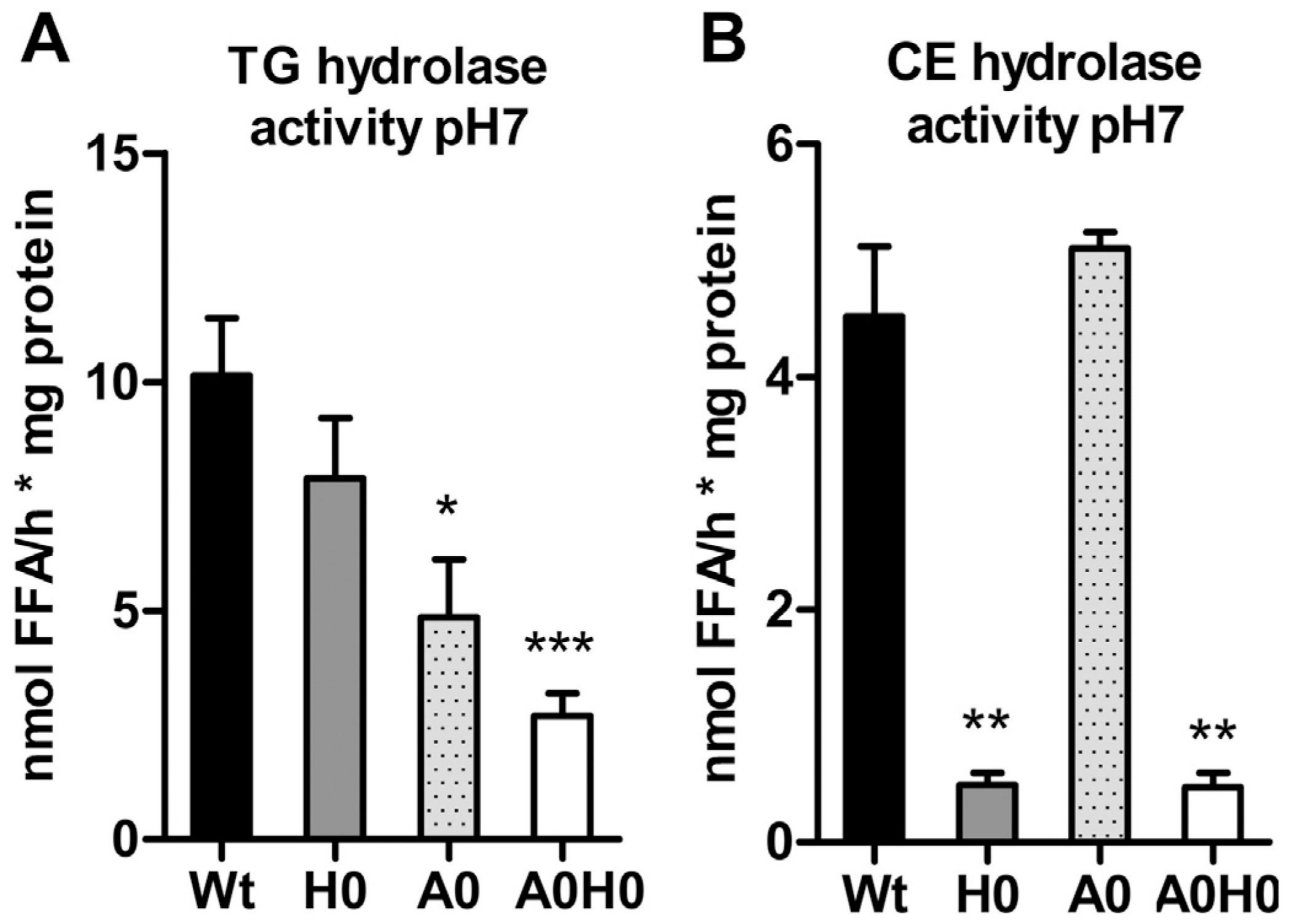
Abolished neutral TG hydrolase activity in *A0H0* macrophages. Neutral (A) TG and (B) CE hydrolase activities were measured in Wt, *H0*, *A0*, and *A0H0* macrophages. Data are presented as mean (n = 4–6) + SEM. *, p < 0.05; **, p ≤ 0.01; ***, p ≤ 0.001.

To elucidate the consequences of ATGL and HSL deficiency in macrophages in vivo, we generated *A0H0* mice. Genotyping results are shown in Supplemental Fig. S1A; lack of HSL and ATGL in macrophages was confirmed by Western blot analysis (Supplemental Fig. S1B). First we determined TG and CE hydrolase activities in macrophage lysates from Wt, *H0*, *A0*, and *A0H0* mice. Neutral TG hydrolase activity was significantly decreased in *A0* and *A0H0* compared with Wt macrophages (51% and 73%, respectively) (Fig. 1A). Neutral CE hydrolase activity was strongly decreased in *H0* (88%) and *A0H0* (90%) compared with Wt and *A0* macrophages (Fig. 1B). These data indicate that the absence of HSL alone markedly contributes to the reduction in neutral CE hydrolase activity, whereas ATGL together with HSL accounts for the main neutral TG hydrolase activities in macrophages.

### 4.2. TG-rich LD accumulation in A0H0 macrophages

**Fig. 2:**
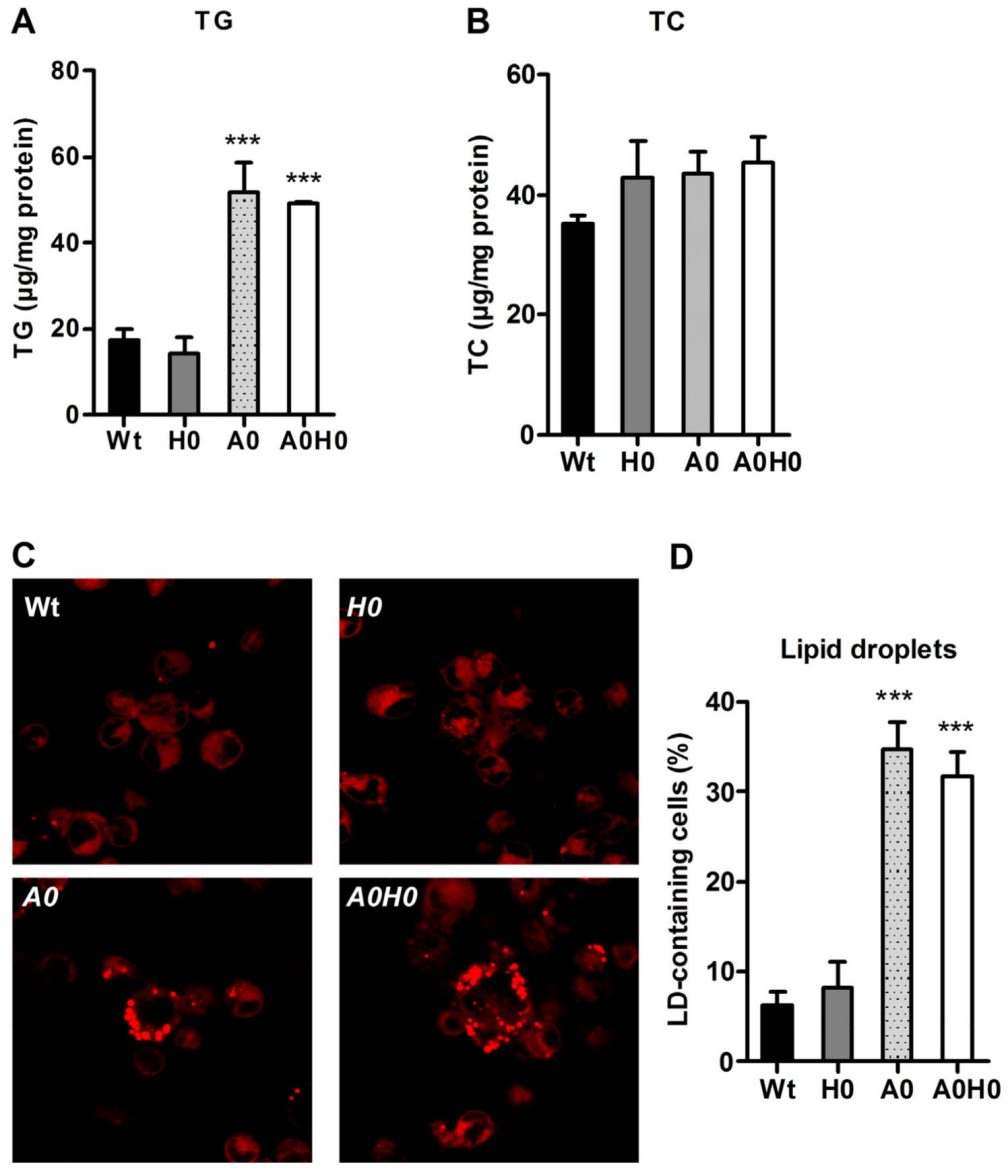
Increased TG concentrations and number of lipid droplets in *A0H0* macrophages. (A) TG and (B) TC concentrations in Wt, *H0*, *A0* and *A0H0* macrophages after lipid extraction were measured enzymatically. Data are presented as mean values (n = 3–6) + SEM. ***, p ≤ 0.001. (C) LDs in macrophages were visualized by Nile red staining. Image magnification, ×63. (D) Quantification of LD-containing macrophages cultivated in DMEM/10% LPDS. Data are presented as the mean percentage of LD-containing cells + SEM from at least 300 cells per group. ***, p ≤ 0.001.

To investigate the rate of LD accumulation in *A0H0* macrophages, we analyzed intracellular TG and cholesterol concentrations. We observed ~3-fold increased TG concentrations in *A0* and *A0H0* macrophages compared to Wt and *H0* macrophages (Fig. 2A). TG content in *H0* and Wt macrophages was comparable, indicating that the absence of HSL failed to affect intracellular TG stores in *A0H0* macrophages (Fig. 2A). In accordance with our previous findings in *H0* [14] and *A0* macrophages [17], TC concentrations were unchanged in *A0H0* compared to Wt macrophages (Fig. 2B). To determine the LD content, we analyzed the cells after Nile red staining by fluorescence microscopy. Macrophages from *A0* and *A0H0* mice had substantially more LDs compared with macrophages from Wt and *H0* mice (Fig. 2C, D). HSL deficiency did not lead to noticeable alterations in the amount of LDs, neither in *A0H0* compared with *A0* nor in *H0* compared with Wt macrophages.

### 4.3. Autophagy is a general recycling mechanism in macrophages

**Fig. 3:**
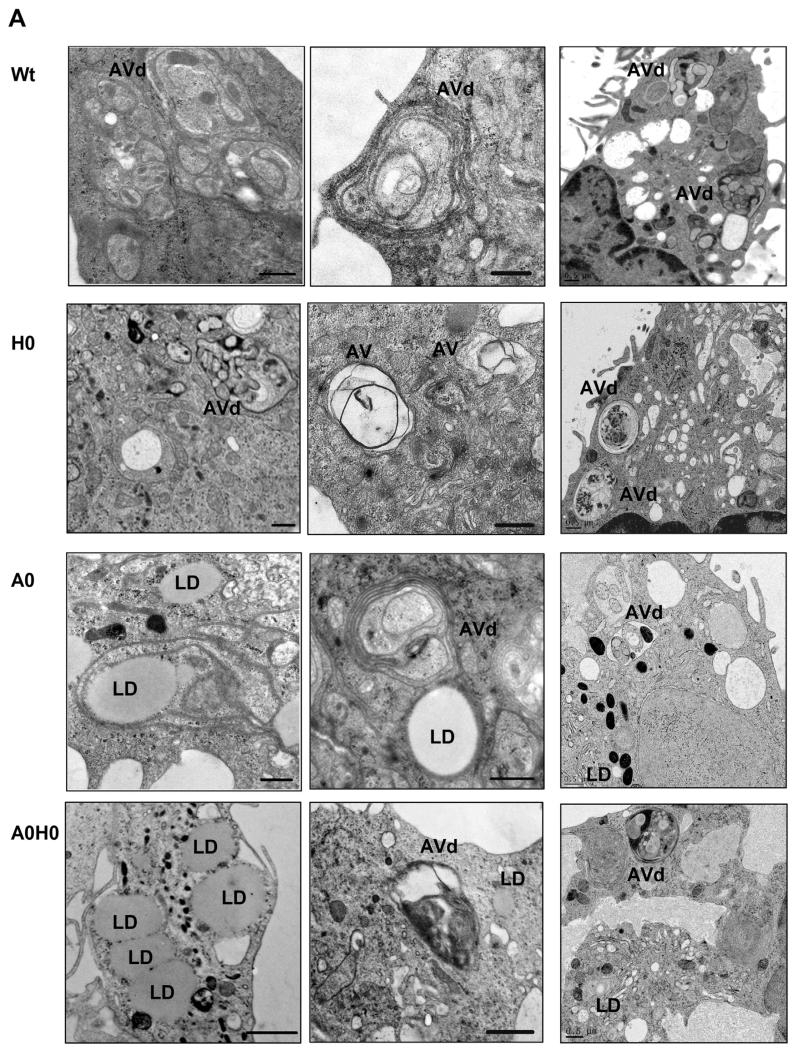

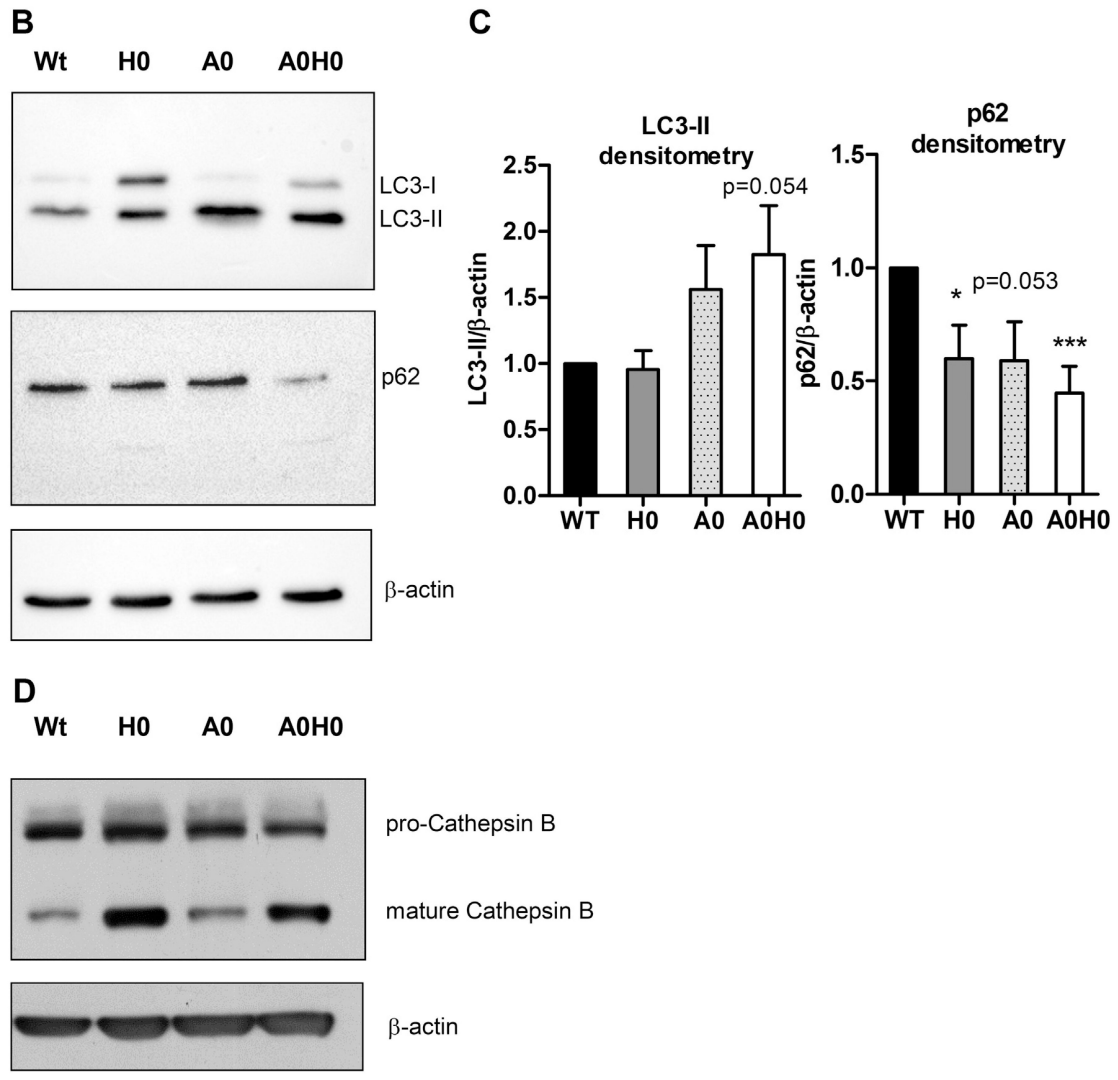
Increased autophagosome formation in *A0H0* macrophages. (A) Representative electron micrographs of Wt, *H0*, *A0*, and *A0H0* macrophages, showing degradative autophagic vacuoles (AVd) in cells from all phenotypes and LDs in *A0* and *A0H0* macrophages. Right panel: Electron micrographs after high-pressure freezing (HPF). Scale bars: 0.5 μm. (B, D) Western blotting of macrophage lysates using (B) anti-LC3B, anti-p62, and (D) anti-cathepsin B specific antibodies. Protein expression of β-actin was determined as loading control. (C) Densito-metric quantification of LC3-II/β-actin (n = 7–11) and p62/β-actin (n = 6–7) of independent experiments + SEM, relative to the expression in Wt macrophages. *, p < 0.05; ***, p ≤ 0.001.

It has been recently shown that autophagy is involved in the regulation of intracellular lipid content [2]. Utilizing electron microscopy we found a substantial amount of structures resembling degradative autophagic vacuoles (AVd) in macrophages from all genotypes (Fig. 3A). In *A0* macrophages, we observed LDs in very close proximity to autophagic vesicle-like structures. Closer inspection revealed that LDs are not enclosed within these membrane structures, which are morphologically not comparable to AVd presented in other images. To investigate whether autophagy might regulate intracellular TG levels in *A0H0* macrophages, we determined LC3 degradation. During autophagy, cytosolic LC3 (LC3-I) is modified to its membrane-bound form (LC3-II) located on pre-autophagosomes and autophagosomes, which makes it a commonly used autophagosome marker [25]. As shown in Fig. 3B, macrophages from all genotypes showed a substantial amount of LC3-II, indicating that autophagy is a general recycling mechanism in macrophages. The ratio of LC3-II to β-actin trended to be increased in *A0* and *A0H0* macrophages compared to controls (Fig. 3C). In addition, we analyzed protein expression of p62, a chaperone that shuttles intracellular protein aggregates into autolysosomes for degradation. Since the entire p62-protein aggregate is degraded after engulfment by the autolysosome, expression levels of p62 are inversely correlated with autophagic flux. Protein expression of p62 trended to be reduced in macrophages from *H0* and *A0* mice, however, this reduction was statistical significant in *A0H0* macrophages (Fig. 3C). Reduced expression of p62 and increased abundance of the mature form of the lysosomal protease cathepsin B (Fig. 3D) in *A0H0* macrophages suggest that autophagy is highly active in macrophages deficient in both ATGL and HSL.

### 4.4. Intact autophagic flux and lysosomal activity in A0H0 macrophages

**Fig. 4:**
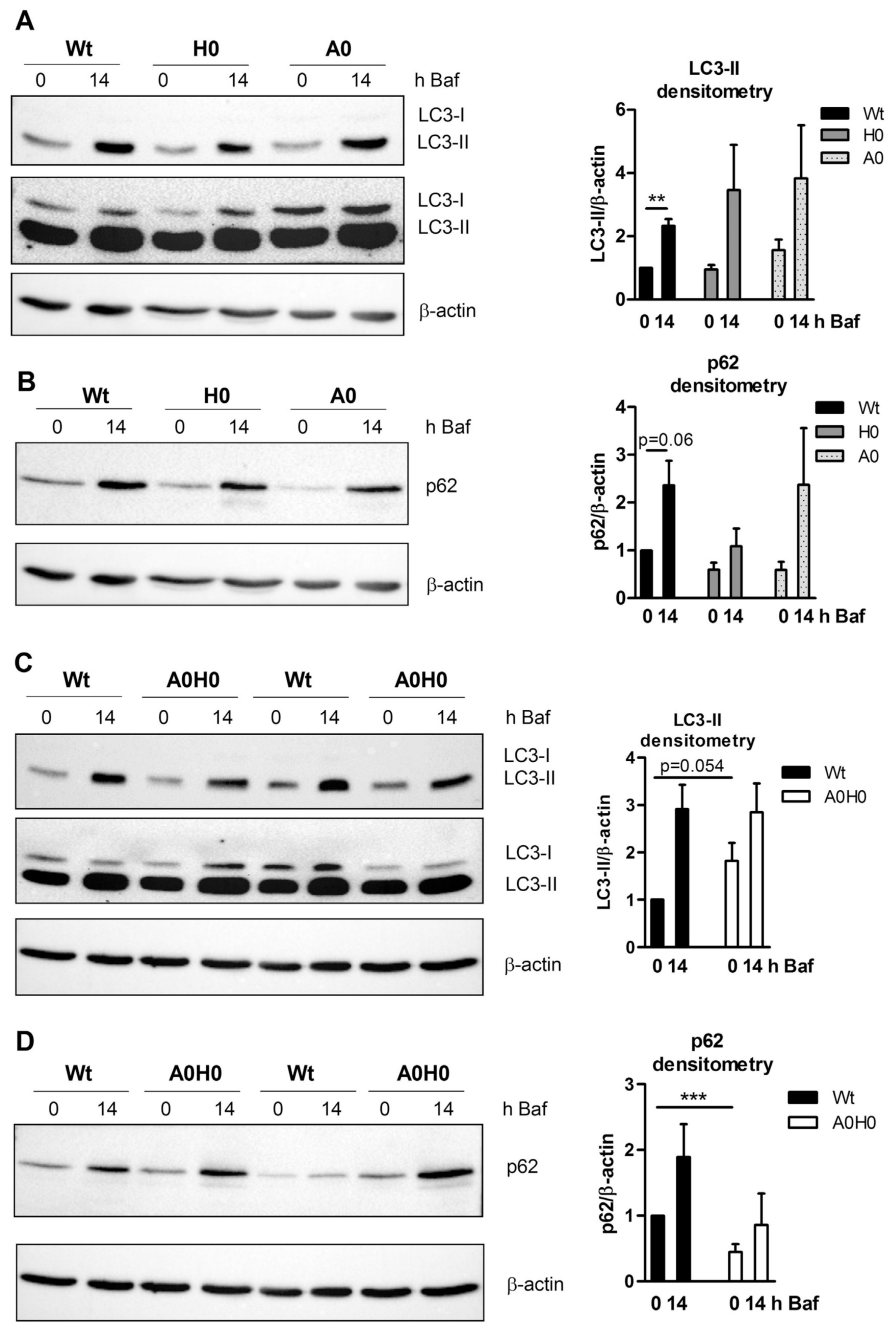

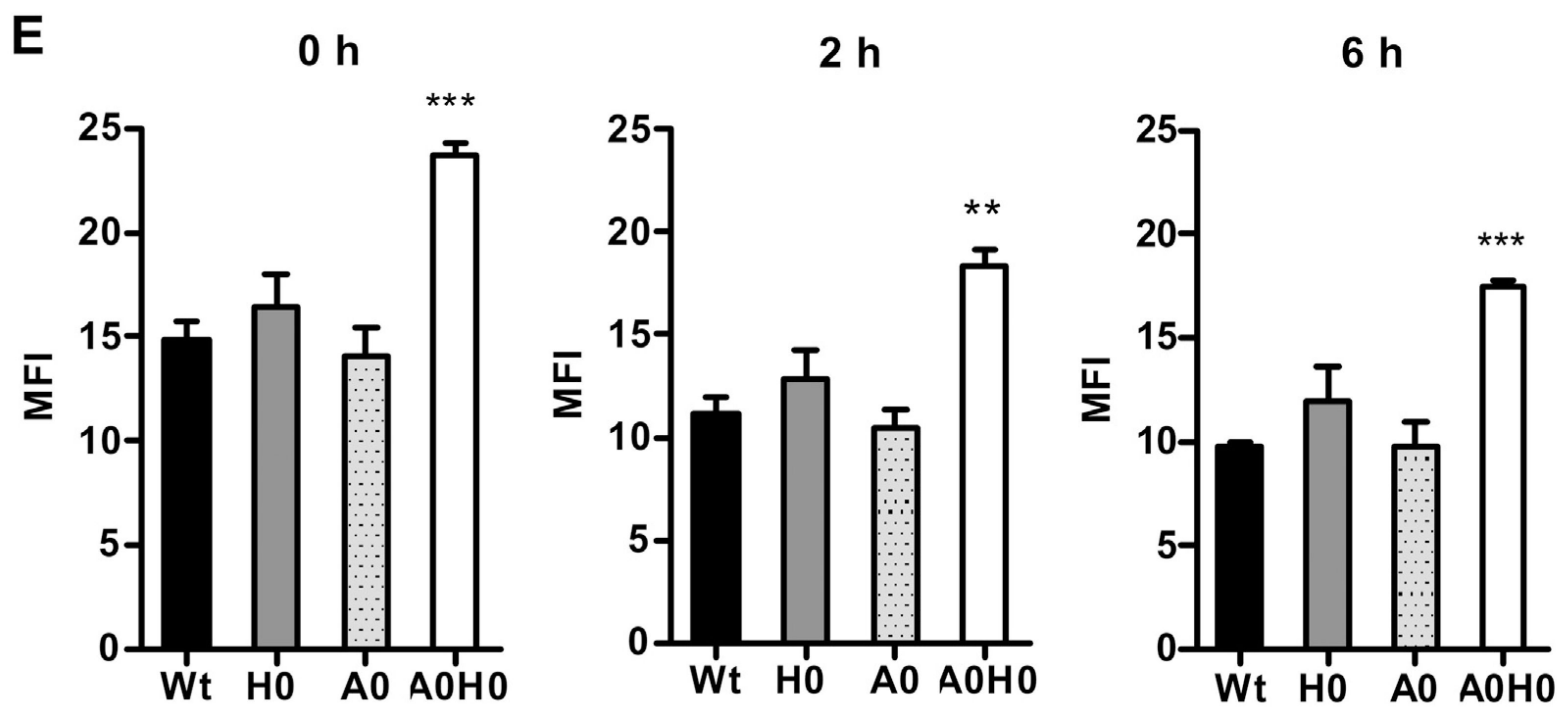
Intact autophagic flux in *A0H0* macrophages. Macrophages were cultured in DMEM/10% LPDS for 24 h. (A–D) Macrophages were incubated with 10 nM bafilomycin A1 (Baf) for 0 and 14 h and assayed for (A, C) LC3 (upper blot: short exposure time to quantify LC3-II; lower blot: longer exposure time to visualize LC3-I) and (B, D) p62 protein expression. Protein expression of β-actin was determined as loading control. Representative Western blot analysis of at least 4 independent experiments is shown. Densitometric quantification of LC3-II/β-actin and p62/β-actin of (A) n = 4–7, (B) n = 4–8, (C) n = 6–10, and (D) n = 5–11 + SEM. (E) Cells were starved for 1 h in HBSS and then incubated with DQ-BSA (final concentration 10 μg/ml) for 15 min at 37 °C. Red-fluorescent DQ-BSA was analyzed by flow cytometry using a FACSCalibur flow cytometer after 0, 2, and 6 h, respectively. Data are presented as mean (n = 3–9) + SEM. **, p ≤ 0.01; ***, p ≤ 0.001.

To analyze the autophagic flux we next incubated the macrophages with bafilomycin A1, which inhibits the fusion between autophagosomes and lysosomes, thereby preventing maturation of autophagic vacuoles and degradation of LC3-II and p62. Bafilomycin A1 treatment resulted in comparable increase in LC3-II (Fig. 4A, C) and p62 (Fig. 4B, D) expression in macrophages from Wt, *A0*, *H0*, and *A0H0* mice with a trend to decreased p62 expression in *H0* (Fig. 4B) and *A0H0* (Fig. 4D) macrophages. Chloroquine treatment resulted in similarly increased protein expression of LC3-II and p62 in Wt and A0H0 macrophages (Supplemental Fig. 2A, B).

In addition, we measured lysosomal activity with another method by treating macrophages with DQ^™^ Red BSA (DQ-BSA) and analyzing the fluorescence by flow cytometry. DQ-BSA is a derivative of BSA that is labeled with a self-quenched red fluorescent dye and dequenched in acidic intracellular lysosomal compartments by lysosomal proteases, releasing red fluorescence [26]. After starving the cells for 1 h and DQ-BSA incubation for 15 min, Wt, *H0*, and *A0* macrophages showed comparable red fluorescence immediately after loading (0 h) and after chasing the cells for 2 h and 6 h (Fig. 4E). In *A0H0* macrophages, we observed significantly increased amounts of dequenched DQ-BSA at all time points (Fig. 4E), suggesting that *A0H0* macrophages have elevated lysosomal proteolytic activity.

### 4.5. Acid TG hydrolase activity is markedly diminished in A0H0 macrophages

**Fig. 5:**
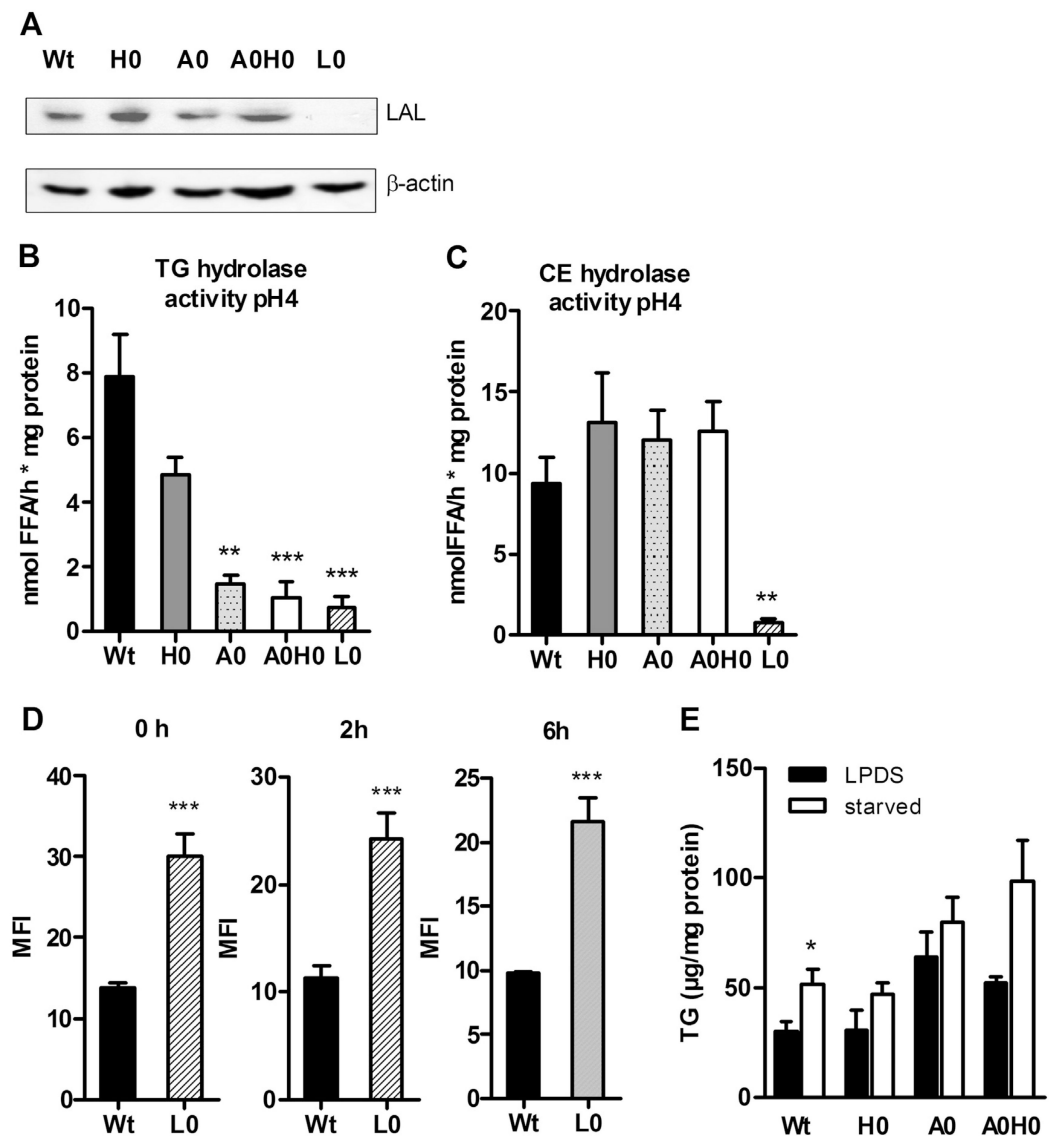
Reduced acid TG hydrolase activity in *A0H0* macrophages. (A) Western blot analysis of LAL protein expression in macrophage lysates. Macrophages from Lal-deficient (*L0*) mice were used as negative control. Protein expression of β-actin was determined as loading control. Acid (B) TG and (C) CE hydrolase activities were measured in Wt, *H0*, *A0*, and *A0H0* macrophages. Macrophages from *L0* mice were used as control. Data are presented as mean (n = 3–8) + SEM. (D) *L0* macrophages were starved for 1 h in HBSS and incubated with 10 μg/ml DQ-BSA for 15 min at 37 °C. Red-fluorescent DQ-BSA was analyzed by flow cytometry after 0, 2, and 6 h, respectively. Data are presented as mean (n = 4–7) + SEM. ***, p ≤ 0.001. (E) Macrophages were incubated in HBSS for 90 min and intracellular TG concentrations were determined. Data are presented as mean + SEM. *, p < 0.05; **, p ≤ 0.01; ***, p ≤ 0.001.

Finally, we investigated whether the terminal step in autophagic lipid degradation is induced in lipase-deficient macrophages. To this extent, we first determined the protein expression of LAL, the major TG and CE hydrolase in lysosomes. As shown in Fig. 5A, LAL protein expression was comparable between macrophages from all genotypes. *Lal-deficient* (L0) macrophages were used as control. Acid TG hydrolase activity, which reveals the activity of the lysosomal TG hydrolase LAL, was slightly decreased in *H0* and ~80% reduced in *A0* and *A0H0* compared to Wt macrophages (Fig. 5B). Acid CE hydrolase activity was comparable in macrophages from all genotypes (Fig. 5C). As a control, *L0* macrophages showed drastically reduced acid TG and CE hydrolase activities (Fig. 5B, C). Comparable with *A0H0* macrophages, *L0* macrophages showed increased dequenched DQ-BSA (Fig. 5D), suggesting an elevated lysosomal proteolysis even in cells lacking the major acid hydrolase. Starvation, a known inducer of autophagy, failed to decrease intracellular TG concentrations in macrophages of all genotypes (Fig. 5E). We starved the cells for 1.5 h since a longer starvation period drives lipase-deficient cells into cell death (unpublished observation). In summary, our data suggest that although autophagy is active in macrophages lacking both ATGL and HSL, degradation of LDs by LAL is less effective.

### 4.6. Bafilomycin A1 treatment does not affect lipid turnover in macrophages

**Fig. 6:**
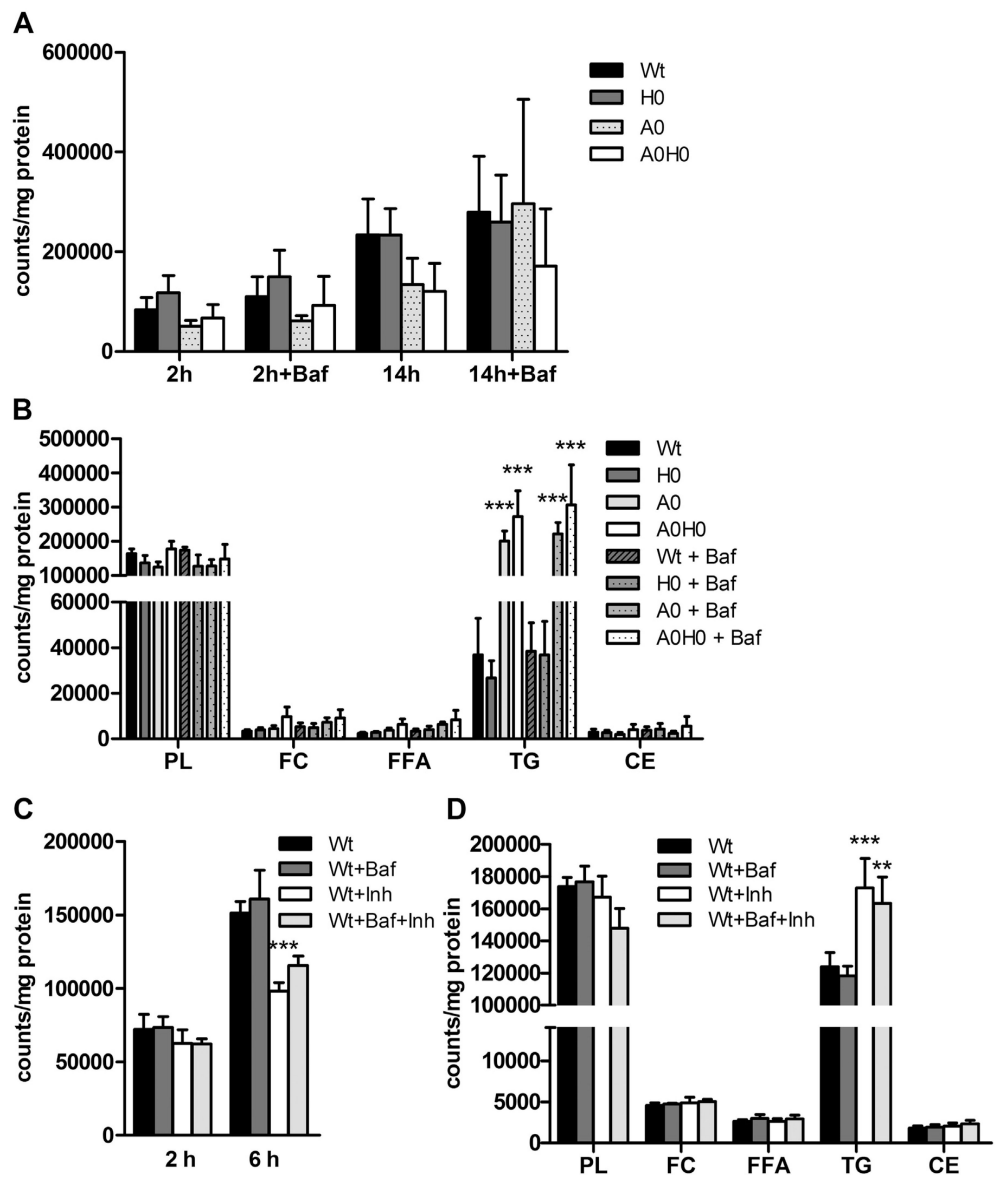

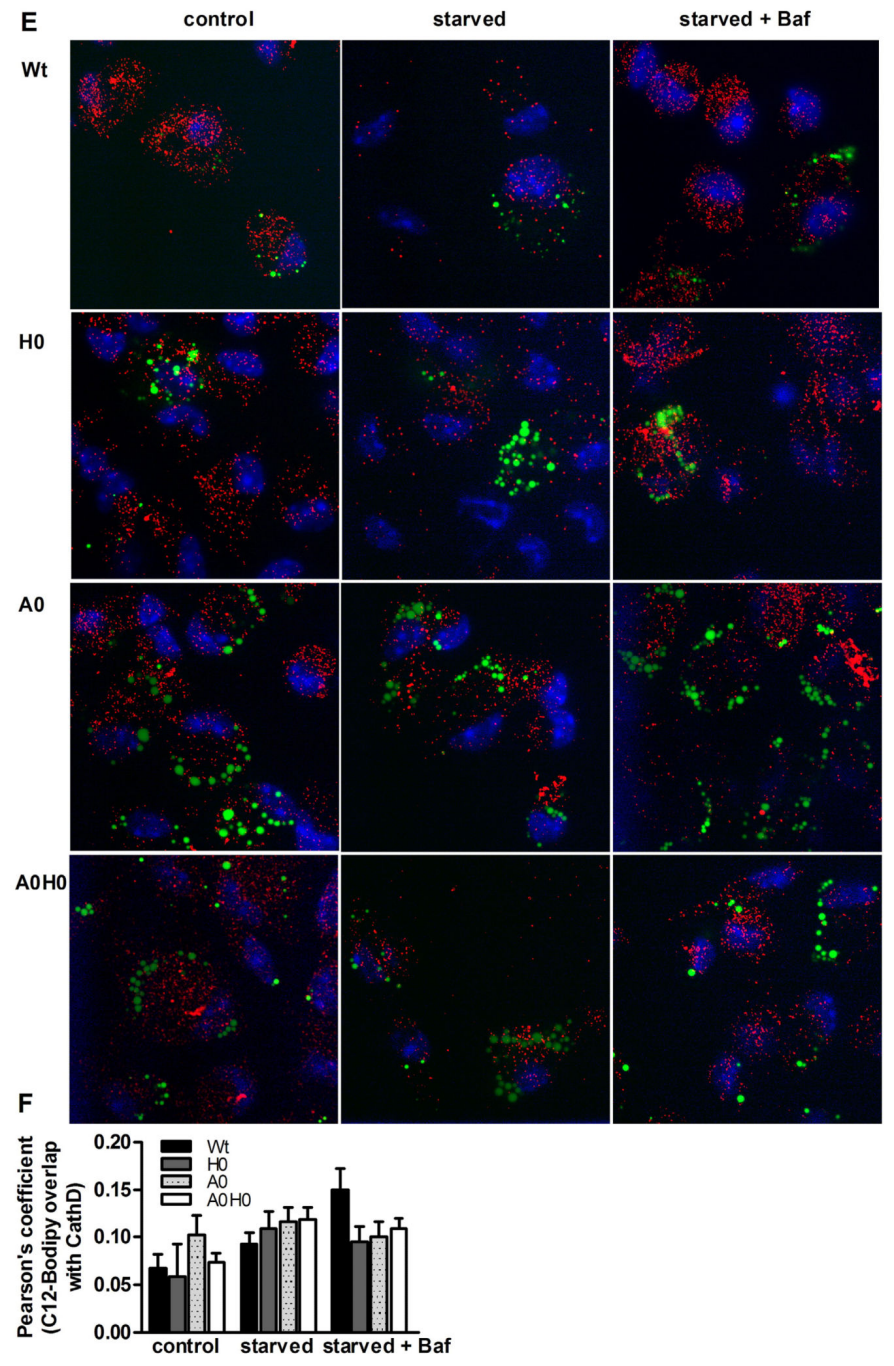
Bafilomycin A1 treatment does not affect lipid turnover in macrophages. Macrophages were cultured in DMEM/1% P/S containing 300 μM OA-BSA and 1 μCi [^3^H]OA-BSA per ml for 24 h. Thereafter, macrophages were incubated with 10 nM bafilomycin A1 (Baf) +/− Atglistatin and HSL-inhibitor for 14 h. (A, C) FA release into the medium and (B, D) intracellular FA incorporation were determined by liquid scintillation counting and normalized to protein content. Data are presented as mean (A, B) (n = 3–5) and (C, D) (n = 5) + SEM compared to Wt +/− Baf. (E) Macrophages were pulsed with C12-BODIPY for 24 h, washed (control), and chased with HBSS (starved) and Baf for 1 h. Lysosomes were labeled using cathepsin D antibody. (F) Relative cellular colocalization of C12-BODIPY with cathepsin D in pools of cells from 2 mice per genotype was quantified by Pearson’s coefficient analysis. Data are expressed as means (n = 7–10 fields/genotype) + SEM. **, p ≤ 0.01; ***, p ≤ 0.001.

To investigate the role of autophagy in LD turnover in macrophages, we loaded macrophages with [^3^H]OA-BSA and analyzed the flux in the absence and presence of bafilomycin A1. In *A0* and *A0H0* macrophages, there was a trend toward decreased FA release into the medium after 14 h of bafilomycin A1 treatment. However, we failed to observe any differences in untreated compared to bafilomycin A1-treated cells (Fig. 6A). Furthermore, we found increased FA incorporation into TGs in *A0* and *A0H0* macrophages, which was unaffected by bafilomycin A1 treatment (Fig. 6B). We observed no differences in incorporation of [^3^H]OA-BSA into other lipid classes between the different genotypes or after bafilomycin A1 incubation. Next, we treated Wt cells with inhibitors against ATGL (Ai) and HSL (Hi), resulting in significantly decreased FA release (Fig. 6C). This finding suggests that the inhibitors efficiently blocked lipolysis. Comparable to the results from *A0* and *A0H0* macrophages, we observed increased FA incorporation into TGs of inhibitor-treated Wt cells, which was unaffected by bafilomycin A1 treatment (Fig. 6D). In summary, results from these pulse-chase experiments revealed that incorporation of FAs into lipid classes was independent of bafilomycin A1 in macrophages from all genotypes.

In analogy, we incubated macrophages with C12-BODIPY and chased the fluorescently labeled FA analogue under starvation conditions in the absence and presence of bafilomycin A1, respectively. To address whether lipophagy is active, we studied the co-localization of C12-BODIPY with lysosomes by immunohistochemistry using cathepsin D as a lysosomal marker. In macrophages from *A0* and *A0H0* mice, we observed a pronounced number of large LDs, which were absent in Wt and *H0* macrophages (Fig. 6E). In macrophages from all genotypes, we found C12-BODIPY to be present in cytosolic LDs without any overlap with lysosomes, independent of bafilomycin A1 treatment (Fig. 6E and F).

## 5. Discussion

TGs are the most abundant form of stored neutral lipid in white adipose and many other tissues. In most tissues, TGs are metabolized during lipolysis by the intracellular lipases ATGL and HSL. In white adipose tissue of *A0* mice, HSL inhibition resulted in more than 95% reduction of neutral TG hydrolase activity [16], indicating that other lipases appear to play only a quantitatively minor role in fat cell lipolysis. We have previously reported that macrophages from *A0* and *H0* mice showed reduced in vitro neutral TG hydrolase activity [14,17]. In contrast to *A0* macrophages, however, *H0* macrophages lack TG accumulation. We generated *A0H0* mice and investigated macrophage lipid metabolism in order to (i) analyze the contribution of ATGL and HSL to TG mobilization in macrophages and to (ii) elucidate if autophagy might be a rescue mechanism to produce energy substrates. In all experiments, macrophages were cultivated in lipoprotein-deficient serum to exclude any effects that may originate from exogenous lipid loading.

Neutral CE hydrolase activity was unchanged in *A0* macrophages but significantly reduced in *H0* and *A0H0* macrophages with an identical decrease in both genotypes, indicating that decreased CE hydrolase activity in *A0H0* macrophages was only due to the lack of HSL. TC concentrations, however, were comparable in macrophages from all genotypes. This finding is in agreement with a previous study, in which we demonstrated lack of CE accumulation in *H0* macrophages [14]. We concluded that additional enzymes must exist that cooperate with HSL to regulate intracellular CE levels in vivo. A candidate responsible for neutral CE hydrolase activity in macrophages is KIAA1363 (neutral CE hydrolase 1, Nceh1) [27].

*H0* macrophages have reduced TG hydrolase activity but failed to accumulate TGs [14]. This result is in accordance with previous data showing DG but no TG accumulation in white adipose tissue of *H0* mice [12]. Reduced TG hydrolase activity and TG accumulation in essentially all cells and tissues of *A0* mice demonstrated that ATGL is crucial for TG hydrolysis [15,17]. Neutral TG hydrolase activity was reduced by 82% in white adipose tissue of *A0* mice [15]. In accordance with [16], we observed markedly reduced TG hydrolase activity and an accumulation of TG-rich LDs in *A0H0* macrophages. Visualization of LDs by electron and fluorescence microscopy revealed a substantial amount of LDs in *A0* and *A0H0* macrophages. Interestingly, however, we observed comparable TG concentrations in *A0H0* and *A0* macrophages. One might speculate that the absence of ATGL results in intracellular TG concentrations that cannot be further increased by the additional lack of HSL. A similar phenomenon was observed in white adipose tissue of *A0H0* mice (data not shown). In *A0* macrophages, HSL contributes to TG hydrolase activity, resulting in reduced but still substantial TG hydrolase activity. Since macrophages lacking both ATGL and HSL show 73% reduced TG hydrolase activity, we conclude that ATGL and HSL are the major cytoplasmic lipases required for TG hydrolysis in macrophages. Candidate enzymes, which are expressed in murine macrophages and might be responsible for the residual TG hydrolase activity in *A0H0* macrophages, are hepatic lipase [28], LPL [29–31], and carboxylesterase (Ces)1d [32]. Lack of hepatic lipase resulted in reduced atherosclerosis in apolipoprotein E- and lecithin:cholesterol acyltransferase-deficient mice [33,34]. This enzyme, however, is only produced by macrophages present in aortic lesions [34] and might therefore only play a minor role as TG hydrolase in peritoneal macrophages. Macrophage expression of LPL promotes foam cell formation and atherosclerosis in vivo [29–31]. Both hepatic lipase and LPL are extracellular lipases, which facilitate the entry of lipids into the cell by the hydrolysis of TG (and phospholipids) in circulating lipoproteins. Thus, these enzymes are unlikely responsible for mobilization of lipids from cytoplasmic LDs. Ces1d (previously called Ces3 or TGH) is mainly expressed in adipose tissue, where it showed little TG hydrolase activity [32]; it is expressed in macrophages at low levels.

During autophagy, autophagosomes fuse with lysosomes and degrade their contents, releasing the breakdown products into the cytosol. Especially during starvation, autophagy contributes to energy and building block supply. Thus, along with lipolysis, autophagy is one of two conserved responses to fasting. Since lipolysis is markedly diminished in *A0H0* macrophages and these cells might have a fasting-like phenotype due to reduced intracellular FA concentrations, we hypothesized that autophagy might be induced. We observed a substantial amount of the autophagy marker LC3-II in macrophages from all geno-types, indicating that autophagy in macrophages is a consistently induced mechanism for degradation of intracellular material. Since the autophagosome is an intermediate structure in the dynamic pathway, the number of autophagosomes observed at any specific time point is the result of the balance between the rate of their generation and their conversion into autolysosomes [35]. For the determination of cellular autophagic activity the measurement of autophagic flux is more reliable although techniques for assessing autophagic flux are limited [35]. We used bafilomycin A1 as a potent and specific inhibitor of vacuolar H^+^-ATPase, which prevents maturation of autophagic vacuoles by inhibiting fusion between autophagosomes and lysosomes [36]. As a result, LC3-II degradation is blocked and its accumulation can be monitored [37]. The differences in the amount of LC3-II between samples in the absence or presence of bafilomycin A1 represent the amount of LC3 that is degraded in lysosomes [38–40]. p62 also accumulates when autophagy is inhibited, and decreased levels can be observed when autophagy is induced [35,41,42]. Comparable with macrophages from all other genotypes, loss of both HSL and ATGL in macrophages resulted in increased protein expression of LC3-II after incubating the cells with bafilomycin A1, indicating an increased number of autophagosomes that cannot be degraded anymore. In analogy, p62 expression was also elevated in macrophages from all genotypes compared to untreated cells. Although not significantly different, the increase in p62 protein expression prior and after bafilomycin A1 treatment was more pronounced in Wt than A0H0 macrophages. Incubation with chloroquine, another inhibitor of autophagy, resulted in comparable p62 expression between Wt and *A0H0* macrophages. Thus, our results indicate that autophagic flux is intact in macrophages with defective lipolysis. p62 does not accumulate in *A0H0* macrophages after treatment with bafilomycin A1 to the same extent as compared to Wt macrophages. A possible explanation might be the one given by Klionsky et al. [43] that a clear correlation between increases in LC3-II and decreases in p62 is sometimes missing. Thus, although analysis of p62 can assist in assessing the impairment of autophagy or autophagy flux, the authors recommend using p62 only in combination with other methods such as LC3-II turnover to monitor flux. In addition, Puissant et al. [44] highlight the controversies and misinterpretations of p62 expression as a marker of autophagic flux because p62 is subject to complex regulation at both the transcriptional and post-translational levels. We therefore used another independent method to measure lysosomal function in macrophages. Dequenching of DQ-BSA is used to monitor the fusion of endosomes and amphisomes with lysosomes, thereby serving as a measure of a functional lysosomal activity [45]. We observed increased amount of dequenched DQ-BSA in *A0H0* macrophages, providing evidence that deficiency of ATGL and HSL in macrophages is not associated with defective lysosomal activity. Together with increased mature cathepsin B expression, these findings rather demonstrate that *A0H0* macrophages have elevated lysosomal proteolytic activity.

Autophagy was previously shown to regulate hepatic lipid stores in vivo [2]. This previously unknown link between autophagy and lipid metabolism (termed lipophagy) was demonstrated by increased TG and DG concentrations in vitro and in vivo, and decreased TG breakdown in autophagy-inhibited conditions. Moreover, it has been shown that components of the autophagic pathway associate with LDs [2]. Ouimet et al. have recently reported that autophagy also contributes to the intracellular breakdown of CE in macrophage-derived foam cells that have been loaded with modified LDL [26]. Cytoplasmic LD-associated CEs delivered to lysosomes are degraded by LAL-dependent lipolysis, thereby generating FC for efflux to extracellular acceptors [26]. Since acid CE hydrolase activity (resembling LAL activity) is comparable between lipase-deficient and Wt macrophages, CE accumulation does not occur, and “fatty lysosomes” as observed in *L0* macrophages (data not shown) are absent in macrophages from cytosolic lipase-deficient mice. Without changes in cytosolic CE content no adaptive response by LAL might take place. Is degradation of cytosolic LDs by lipophagy an active mechanism for generating FAs as energy substrate in *A0H0* macrophages? Markedly reduced acid TG hydrolase activity in *A0* and *A0H0* macrophages argue against this hypothesis. Unaltered LAL protein content despite decreased TG hydrolase activity in *A0* and *A0H0* macrophages may imply a distinct regulation of TG and CE hydrolysis by LAL, suggesting a possible involvement of unknown co-activator(s). To assess whether inhibition of autophagy affects turnover of lipids stored in LDs we performed lipid flux experiments in the absence and presence of bafilomycin A1. Treatment with bafilomycin A1, however, failed to affect FA incorporation, metabolism and release, indicating that inhibition of autophagy does not mediate turnover of LDs in macrophages. Even under conditions of defective lipolysis, inhibition of autophagy did not reduce lipid turnover, indicating that peritoneal macrophages do not rely on autophagic LD degradation.

## 6. Conclusion

Our data provide conclusive evidence that ATGL-mediated TG breakdown is the crucial step of TG hydrolysis in macrophages. We demonstrate that loss of ATGL reaches a maximal TG accumulation capacity in macrophages, resulting in comparable intracellular TG levels between *A0* and *A0H0* macrophages. Increased cathepsin B abundance together with increased LC3-II and reduced p62 protein expression and increased dequenching of DQ-BSA suggest increased autophagic flux and lysosomal proteolysis in *A0H0* macrophages. In fact, we observed that starvation-induced autophagy did not decrease but rather increase intracellular TG content in macrophages. This is in consistence with a recent study of FA trafficking under nutrient deprivation in mouse embryonic fibroblasts [5]. The authors demonstrated that FAs generated by autophagy replenish intracellular LDs and that FAs used for mitochondrial respiration have to be mobilized by intracellular lipases such as ATGL and HSL. Results from this study together with our findings suggest that autophagy and lipolysis are not necessarily alternative but co-dependent mechanisms to fuel cellular metabolic needs. In *A0H0* macrophages, autophagy but not lipophagy might be triggered as a compensatory mechanism to degrade proteins and cell organelles to circumvent and balance the reduced availability of energy substrates.

## Supplementary Material

1

## Abbreviations

ATGL: adipose triglyceride lipase
CE: cholesteryl ester
DG: diacylglycerol
FA: fatty acid
FC: free cholesterol
HSL: hormone-sensitive lipase
LAL: lysosomal acid lipase
LC3: microtubule-associated protein light chain 3
LD: lipid droplet
LPDS: lipoprotein-deficient serum
OA: oleic acid
TC: total cholesterol
TG: triglyceride

## References

[1] Levine B, Kroemer G (2008). Autophagy in the pathogenesis of disease. Cell.

[2] Singh R, Kaushik S, Wang Y, Xiang Y, Novak I, Komatsu M, Tanaka K, Cuervo AM, Czaja MJ (2009). Autophagy regulates lipid metabolism. Nature.

[3] Singh R, Xiang Y, Wang Y, Baikati K, Cuervo AM, Luu YK, Tang Y, Pessin JE, Schwartz GJ, Czaja MJ (2009). Autophagy regulates adipose mass and differentiation in mice. J. Clin. Investig.

[4] Zechner R, Zimmermann R, Eichmann TO, Kohlwein SD, Haemmerle G, Lass A, Madeo F (2012). FAT SIGNALS—lipases and lipolysis in lipid metabolism and signaling. Cell Metab.

[5] Rambold AS, Cohen S, Lippincott-Schwartz J (2015). Fatty acid trafficking in starved cells: regulation by lipid droplet lipolysis, autophagy, and mitochondrial fusion dynamics. Dev. Cell.

[6] Li AC, Glass CK (2002). The macrophage foam cell as a target for therapeutic intervention. Nat. Med.

[7] Ross R (1993). The pathogenesis of atherosclerosis: a perspective for the 1990s. Nature.

[8] Jenkins CM, Mancuso DJ, Yan W, Sims HF, Gibson B, Gross RW (2004). Identification, cloning, expression, and purification of three novel human calcium-independent phospholipase A2 family members possessing triacylglycerol lipase and acylglycerol transacylase activities. J. Biol. Chem.

[9] Villena JA, Roy S, Sarkadi-Nagy E, Kim KH, Sul HS (2004). Desnutrin, an adipocyte gene encoding a novel patatin domain-containing protein, is induced by fasting and glucocorticoids: ectopic expression of desnutrin increases triglyceride hydrolysis. J. Biol. Chem.

[10] Zimmermann R, Strauss JG, Haemmerle G, Schoiswohl G, Birner-Gruenberger R, Riederer M, Lass A, Neuberger G, Eisenhaber F, Hermetter A, Zechner R (2004). Fat mobilization in adipose tissue is promoted by adipose triglyceride lipase. Science.

[11] Fredrikson G, Stralfors P, Nilsson NO, Belfrage P (1981). Hormone-sensitive lipase of rat adipose tissue. Purification and some properties. J. Biol. Chem.

[12] Haemmerle G, Zimmermann R, Hayn M, Theussl C, Waeg G, Wagner E, Sattler W, Magin TM, Wagner EF, Zechner R (2002). Hormone-sensitive lipase deficiency in mice causes diglyceride accumulation in adipose tissue, muscle, and testis. J. Biol. Chem.

[13] Yeaman SJ (1990). Hormone-sensitive lipase—a multipurpose enzyme in lipid metabolism. Biochim. Biophys. Acta.

[14] Buchebner M, Pfeifer T, Rathke N, Chandak PG, Lass A, Schreiber R, Kratzer A, Zimmermann R, Sattler W, Koefeler H, Froehlich E, Kostner GM, Birner-Gruenberger R, Chiang KP, Haemmerle G, Zechner R, Levak-Frank S, Cravatt BF, Kratky D (2010). Cholesteryl ester hydrolase activity is abolished in HSL−/− macrophages but unchanged in macrophages lacking KIAA1363. J. Lipid Res.

[15] Haemmerle G, Lass A, Zimmermann R, Gorkiewicz G, Meyer C, Rozman J, Heldmaier G, Maier R, Theussl C, Eder S, Kratky D, Wagner EF, Klingenspor M, Hoefler G, Zechner R (2006). Defective lipolysis and altered energy metabolism in mice lacking adipose triglyceride lipase. Science.

[16] Schweiger M, Schreiber R, Haemmerle G, Lass A, Fledelius C, Jacobsen P, Tornqvist H, Zechner R, Zimmermann R (2006). Adipose triglyceride lipase and hormone-sensitive lipase are the major enzymes in adipose tissue triacylglycerol catabolism. J. Biol. Chem.

[17] Chandak PG, Radovic B, Aflaki E, Kolb D, Buchebner M, Frohlich E, Magnes C, Sinner F, Haemmerle G, Zechner R, Tabas I, Levak-Frank S, Kratky D (2010). Efficient phagocytosis requires triacylglycerol hydrolysis by adipose triglyceride lipase. J. Biol. Chem.

[18] Aflaki E, Balenga NA, Luschnig-Schratl P, Wolinski H, Povoden S, Chandak PG, Bogner-Strauss JG, Eder S, Konya V, Kohlwein SD, Heinemann A, Kratky D (2011). Impaired Rho GTPase activation abrogates cell polarization and migration in macrophages with defective lipolysis. Cell. Mol. Life Sci.

[19] Aflaki E, Radovic B, Chandak PG, Kolb D, Eisenberg T, Ring J, Fertschai I, Uellen A, Wolinski H, Kohlwein SD, Zechner R, Levak-Frank S, Sattler W, Graier WF, Malli R, Madeo F, Kratky D (2011). Triacylglycerol accumulation activates the mitochondrial apoptosis pathway in macrophages. J. Biol. Chem.

[20] Aflaki E, Doddapattar P, Radovic B, Povoden S, Kolb D, Vujic N, Wegscheider M, Koefeler H, Hornemann T, Graier WF, Malli R, Madeo F, Kratky D (2012). C16 ceramide is crucial for triacylglycerol-induced apoptosis in macrophages. Cell Death Dis.

[21] Lammers B, Chandak PG, Aflaki E, Van Puijvelde GH, Radovic B, Hildebrand RB, Meurs I, Out R, Kuiper J, Van Berkel TJ, Kolb D, Haemmerle G, Zechner R, Levak-Frank S, Van Eck M, Kratky D (2011). Macrophage adipose triglyceride lipase deficiency attenuates atherosclerotic lesion development in low-density lipoprotein receptor knockout mice. Arterioscler. Thromb. Vasc. Biol.

[22] Goeritzer M, Schlager S, Radovic B, Madreiter CT, Rainer S, Thomas G, Lord CC, Sacks J, Brown AL, Vujic N, Obrowsky S, Sachdev V, Kolb D, Chandak PG, Graier WF, Sattler W, Brown JM, Kratky D (2014). Deletion of CGI-58 or adipose triglyceride lipase differently affects macrophage function and atherosclerosis. J. Lipid Res.

[23] Mayer N, Schweiger M, Romauch M, Grabner GF, Eichmann TO, Fuchs E, Ivkovic J, Heier C, Mrak I, Lass A, Hofler G, Fledelius C, Zechner R, Zimmermann R, Breinbauer R (2013). Development of small-molecule inhibitors targeting adipose triglyceride lipase. Nat. Chem. Biol.

[24] Bolte S, Cordelieres FP (2006). A guided tour into subcellular colocalization analysis in light microscopy. J. Microsc.

[25] Kabeya Y, Mizushima N, Ueno T, Yamamoto A, Kirisako T, Noda T, Kominami E, Ohsumi Y, Yoshimori T (2000). LC3, a mammalian homologue of yeast Apg8p, is localized in autophagosome membranes after processing. EMBO J.

[26] Ouimet M, Franklin V, Mak E, Liao X, Tabas I, Marcel YL (2011). Autophagy regulates cholesterol efflux from macrophage foam cells via lysosomal acid lipase. Cell Metab.

[27] Okazaki H, Igarashi M, Nishi M, Sekiya M, Tajima M, Takase S, Takanashi M, Ohta K, Tamura Y, Okazaki S, Yahagi N, Ohashi K, Amemiya-Kudo M, Nakagawa Y, Nagai R, Kadowaki T, Osuga J, Ishibashi S (2008). Identification of neutral cholesterol ester hydrolase, a key enzyme removing cholesterol from macrophages. J. Biol. Chem.

[28] Gonzalez-Navarro H, Nong Z, Freeman L, Bensadoun A, Peterson K, Santamarina-Fojo S (2002). Identification of mouse and human macrophages as a site of synthesis of hepatic lipase. J. Lipid Res.

[29] Babaev VR, Fazio S, Gleaves LA, Carter KJ, Semenkovich CF, Linton MF (1999). Macrophage lipoprotein lipase promotes foam cell formation and atherosclerosis in vivo. J. Clin. Investig.

[30] Babaev VR, Patel MB, Semenkovich CF, Fazio S, Linton MF (2000). Macrophage lipoprotein lipase promotes foam cell formation and atherosclerosis in low density lipoprotein receptor-deficient mice. J. Biol. Chem.

[31] Van Eck M, Zimmermann R, Groot PH, Zechner R, Van Berkel TJ (2000). Role of macrophage-derived lipoprotein lipase in lipoprotein metabolism and atherosclerosis. Arterioscler. Thromb. Vasc. Biol.

[32] Soni KG, Lehner R, Metalnikov P, O’Donnell P, Semache M, Gao W, Ashman K, Pshezhetsky AV, Mitchell GA (2004). Carboxylesterase 3 (EC 3.1.1.1) is a major adipocyte lipase. J. Biol. Chem.

[33] Mezdour H, Jones R, Dengremont C, Castro G, Maeda N (1997). Hepatic lipase deficiency increases plasma cholesterol but reduces susceptibility to atherosclerosis in apolipoprotein E-deficient mice. J. Biol. Chem.

[34] Nong Z, Gonzalez-Navarro H, Amar M, Freeman L, Knapper C, Neufeld EB, Paigen BJ, Hoyt RF, Fruchart-Najib J, Santamarina-Fojo S (2003). Hepatic lipase expression in macrophages contributes to atherosclerosis in apoE-deficient and LCAT-transgenic mice. J. Clin. Investig.

[35] Mizushima N, Yoshimori T, Levine B (2010). Methods in mammalian autophagy research. Cell.

[36] Yamamoto A, Tagawa Y, Yoshimori T, Moriyama Y, Masaki R, Tashiro Y (1998). Bafilomycin A1 prevents maturation of autophagic vacuoles by inhibiting fusion between autophagosomes and lysosomes in rat hepatoma cell line, H-4-II-E cells. Cell Struct. Funct.

[37] Tanida I, Minematsu-Ikeguchi N, Ueno T, Kominami E (2005). Lysosomal turnover, but not a cellular level, of endogenous LC3 is a marker for autophagy. Autophagy.

[38] Klionsky DJ, Abeliovich H, Agostinis P, Agrawal DK, Aliev G, Askew DS, Baba M, Baehrecke EH, Bahr BA, Ballabio A, Bamber BA, Bassham DC, Bergamini E, Bi X, Biard-Piechaczyk M, Blum JS, Bredesen DE, Brodsky JL, Brumell JH, Brunk UT, Bursch W, Camougrand N, Cebollero E, Cecconi F, Chen Y, Chin LS, Choi A, Chu CT, Chung J, Clarke PG, Clark RS, Clarke SG, Clave C, Cleveland JL, Codogno P, Colombo MI, Coto-Montes A, Cregg JM, Cuervo AM, Debnath J, Demarchi F, Dennis PB, Dennis PA, Deretic V, Devenish RJ, Di Sano F, Dice JF, Difiglia M, Dinesh-Kumar S, Distelhorst CW, Djavaheri-Mergny M, Dorsey FC, Droge W, Dron M, Dunn WA, Duszenko M, Eissa NT, Elazar Z, Esclatine A, Eskelinen EL, Fesus L, Finley KD, Fuentes JM, Fueyo J, Fujisaki K, Galliot B, Gao FB, Gewirtz DA, Gibson SB, Gohla A, Goldberg AL, Gonzalez R, Gonzalez-Estevez C, Gorski S, Gottlieb RA, Haussinger D, He YW, Heidenreich K, Hill JA, Hoyer-Hansen M, Hu X, Huang WP, Iwasaki A, Jaattela M, Jackson WT, Jiang X, Jin S, Johansen T, Jung JU, Kadowaki M, Kang C, Kelekar A, Kessel DH, Kiel JA, Kim HP, Kimchi A, Kinsella TJ, Kiselyov K, Kitamoto K, Knecht E (2008). Guidelines for the use and interpretation of assays for monitoring autophagy in higher eukaryotes. Autophagy.

[39] Mizushima N, Yoshimori T (2007). How to interpret LC3 immunoblotting. Autophagy.

[40] Rubinsztein DC, Cuervo AM, Ravikumar B, Sarkar S, Korolchuk V, Kaushik S, Klionsky DJ (2009). In search of an “autophagomometer”. Autophagy.

[41] Bjorkoy G, Lamark T, Pankiv S, Overvatn A, Brech A, Johansen T (2009). Monitoring autophagic degradation of p62/SQSTM1. Methods Enzymol.

[42] Komatsu M, Waguri S, Koike M, Sou YS, Ueno T, Hara T, Mizushima N, Iwata J, Ezaki J, Murata S, Hamazaki J, Nishito Y, Iemura S, Natsume T, Yanagawa T, Uwayama J, Warabi E, Yoshida H, Ishii T, Kobayashi A, Yamamoto M, Yue Z, Uchiyama Y, Kominami E, Tanaka K (2007). Homeostatic levels of p62 control cytoplasmic inclusion body formation in autophagy-deficient mice. Cell.

[43] Klionsky DJ, Abdalla FC, Abeliovich H, Abraham RT, Acevedo-Arozena A, Adeli K, Agholme L, Agnello M, Agostinis P, Aguirre-Ghiso JA, Ahn HJ, Ait-Mohamed O, Ait-Si-Ali S, Akematsu T, Akira S, Al-Younes HM, Al-Zeer MA, Albert ML, Albin RL, Alegre-Abarrategui J, Aleo MF, Alirezaei M, Almasan A, Almonte-Becerril M, Amano A, Amaravadi R, Amarnath S, Amer AO, Andrieu-Abadie N, Anantharam V, Ann DK, Anoopkumar-Dukie S, Aoki H, Apostolova N, Arancia G, Aris JP, Asanuma K, Asare NY, Ashida H, Askanas V, Askew DS, Auberger P, Baba M, Backues SK, Baehrecke EH, Bahr BA, Bai XY, Bailly Y, Baiocchi R, Baldini G, Balduini W, Ballabio A, Bamber BA, Bampton ET, Banhegyi G, Bartholomew CR, Bassham DC, Bast RC, Batoko H, Bay BH, Beau I, Bechet DM, Begley TJ, Behl C, Behrends C, Bekri S, Bellaire B, Bendall LJ, Benetti L, Berliocchi L, Bernardi H, Bernassola F, Besteiro S, Bhatia-Kissova I, Bi X, Biard-Piechaczyk M, Blum JS, Boise LH, Bonaldo P, Boone DL, Bornhauser BC, Bortoluci KR, Bossis I, Bost F, Bourquin JP, Boya P, Boyer-Guittaut M, Bozhkov PV, Brady NR, Brancolini C, Brech A, Brenman JE, Brennand A, Bresnick EH, Brest P, Bridges D, Bristol ML, Brookes PS, Brown EJ, Brumell JH (2012). Guidelines for the use and interpretation of assays for monitoring autophagy. Autophagy.

[44] Puissant A, Fenouille N, Auberger P (2012). When autophagy meets cancer through p62/SQSTM1. Am. J. Cancer Res.

[45] Ha SD, Ham B, Mogridge J, Saftig P, Lin S, Kim SO (2010). Cathepsin B-mediated autophagy flux facilitates the anthrax toxin receptor 2-mediated delivery of anthrax lethal factor into the cytoplasm. J. Biol. Chem.

