# Supplementary material for: Active autophagy but not lipophagy in macrophages with defective lipolysis
Source: Biochim Biophys Acta. Author manuscript; Available in PMC 2015 Oct 1. (PMC4562370; doi:10.1016/j.bbalip.2015.06.005)
Supplement: 1 [file NIHMS64441-supplement-1.pdf]

Supplemental figures

Active autophagy but not lipophagy in macrophages with defective lipolysis by Goeritzer, Vujic *et al*

Figure S1

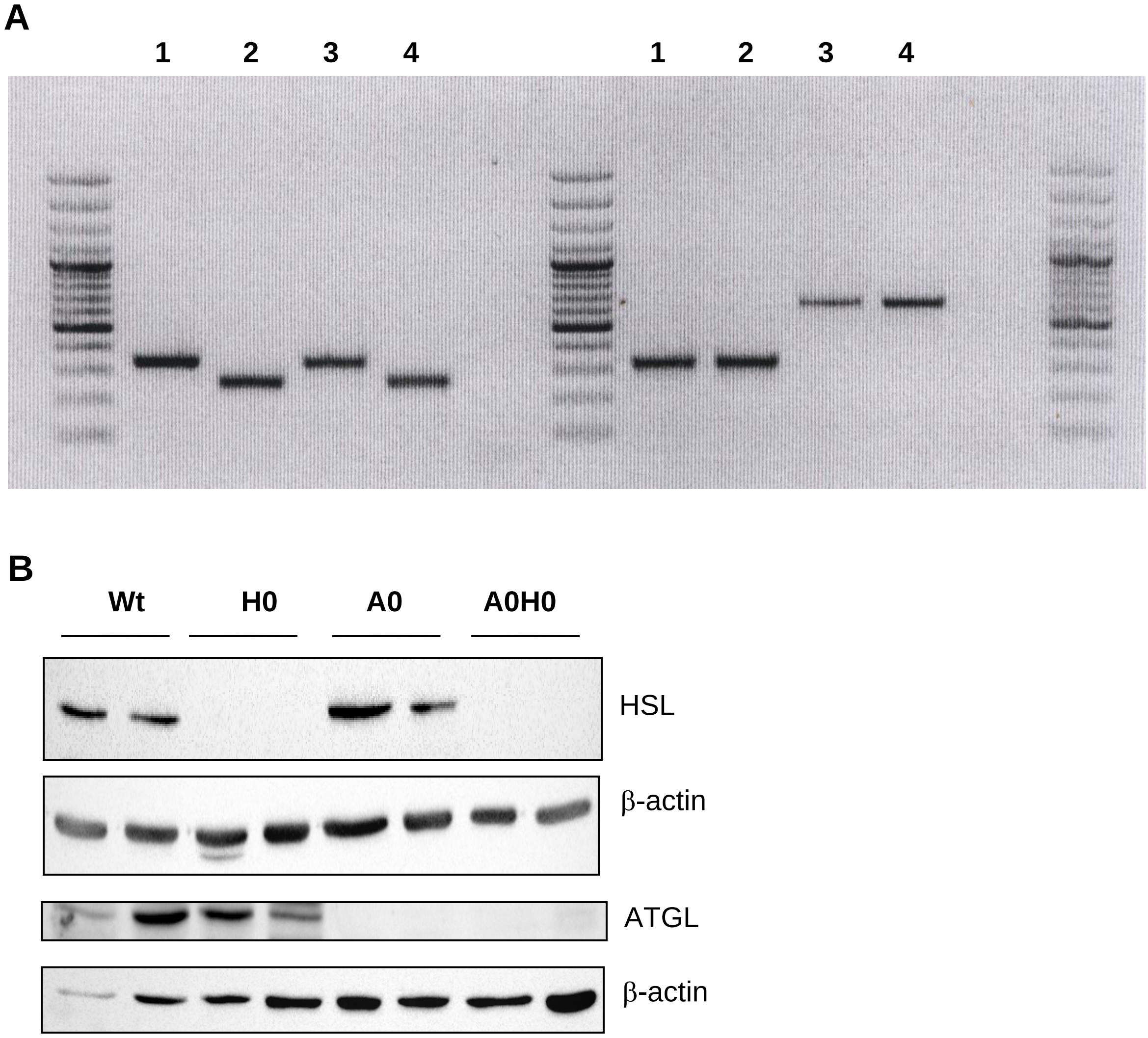

Fig. S1: Genotyping of Wt, *H0*, *A0* and *A0H0* mice and Western blot analysis in macrophages. (A) PCR products of HSL (left side) and ATGL PCR (right side) are shown. The bands represent PCR products of Wt (lane 1), *H0* (lane 2), *A0* (lane 3), and *A0H0* (lane 4) mice. (B) Western blotting of macrophages using anti-ATGL and anti-HSL-specific antibodies. The expression of β-actin was determined as loading control.

Figure S2

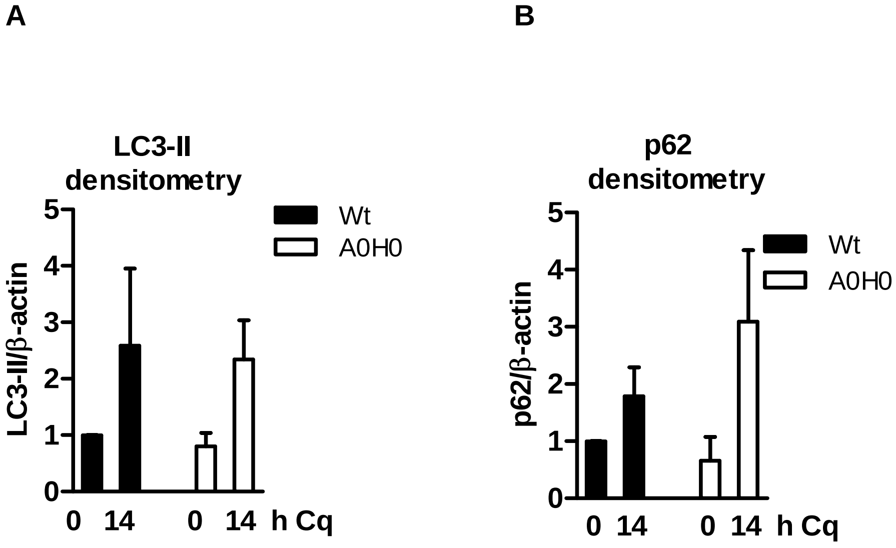

Figure S2: Intact autophagic flux in *A0H0* macrophages. Macrophages were cultured in DMEM/10% LPDS for 24 h. Macrophages were incubated with 30 µM chloroquine (Cq) for 0 and 14 h and assayed for (A)) LC3 and (B) p62 protein expression. Protein expression of β-actin was determined as loading control. Data are presented as densitometric quantification of LC3-II/β-actin and p62/β-actin as mean (n=3) + SEM.
